# SiMPl‐GS: Advancing Cell Line Development via Synthetic Selection Marker for Next‐Generation Biopharmaceutical Production

**DOI:** 10.1002/advs.202405593

**Published:** 2024-08-06

**Authors:** Chansik Yoon, Eun‐ji Lee, Dongil Kim, Siyun Joung, Yujin Kim, Heungchae Jung, Yeon‐Gu Kim, Gyun Min Lee

**Affiliations:** ^1^ Department of Biological Sciences KAIST Daejeon 34141 Republic of Korea; ^2^ Biotherapeutics Translational Research Center KRIBB Daejeon 34113 Republic of Korea; ^3^ Department of Bioprocess Engineering, KRIBB School of Biotechnology UST Daejeon 34141 Republic of Korea; ^4^ BIO Center Daejeon Technopark Daejeon 34054 Republic of Korea

**Keywords:** antibody production, cell line development, Chinese hamster ovary cells, glutamine synthase, selection marker, split intein, synthetic biology

## Abstract

Rapid and efficient cell line development (CLD) process is essential to expedite therapeutic protein development. However, the performance of widely used glutamine‐based selection systems is limited by low selection efficiency, stringency, and the inability to select multiple genes. Therefore, an AND‐gate synthetic selection system is rationally designed using split intein‐mediated protein ligation of glutamine synthetase (GS) (SiMPl‐GS). Split sites of the GS are selected using a computational approach and validated with GS‐knockout Chinese hamster ovary cells for their potential to enable cell survival in a glutamine‐free medium. In CLD, SiMPl‐GS outperforms the wild‐type GS by selectively enriching high producers. Unlike wild‐type GS, SiMPl‐GS results in cell pools in which most cells produce high levels of therapeutic proteins. Harnessing orthogonal split intein pairs further enables the selection of four plasmids with a single selection, streamlining multispecific antibody‐producing CLD. Taken together, SiMPl‐GS is a simple yet effective means to expedite CLD for therapeutic protein production.

## Graphical abstract

An AND‐gate synthetic SiMPl‐GS selection system is developed using split intein‐mediated protein ligation of GS. The SiMPl‐GS system can be directly adopted in GS‐knockout host cells by exploiting the auxotrophic GS selection machinery. Multiple SiMPl interfaces can be applied for multiple‐plasmid selection. SiMPl‐GS and multiple Quad selections have a significant potential for generating cell lines producing bispecific antibodies.

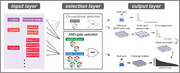

## 1 Introduction

The development of therapeutic proteins is pivotal in modern medicine and offers innovative treatments for various diseases. These proteins are predominantly produced by mammalian cells, mainly the Chinese hamster ovary (CHO) cells.^[^ ^1^ ^]^ As the clinical benefits of complex therapeutic proteins, such as multispecific antibodies, become more pronounced, the demand for efficient cell line development (CLD) has increased. Recent trends in drug discovery underscore the need for rapid and substantial protein production in the early stages of CLD, particularly for investigational new drug (IND)‐enabling toxicology studies.^[^ ^2^, ^3^, ^4^ ^]^ This demand is particularly evident in public health emergencies, such as the coronavirus disease 2019 (COVID‐19) pandemic, where there is an urgent requirement for the early‐stage production of candidate proteins to expedite drug discovery.^[^ ^5^, ^6^, ^7^ ^]^ However, glutamine synthetase (GS)‐based selection, which is a widely used auxotrophic metabolic selection system for therapeutic protein‐producing CLD, presents significant challenges.^[^ ^8^ ^]^ Given that glutamine can be transported in and out of cells through glutamine transporters,^[^ ^9^ ^]^ effectively eliminating low/non‐producers from recombinant cell pools is challenging. Consequently, only a small minority of the cells exhibit high production capabilities. To isolate high producers, an auxiliary minipool selection process is often used, which complicates and prolongs the CLD process to several months to obtain high producers.^[^ ^10^ ^]^ With recent advances in high‐throughput technologies, GS knockout (GS‐KO) bulk pool selection can be successfully used without minipool selection.^[^ ^11^, ^12^ ^]^ Another limitation arises when complex therapeutic protein‐producing cells are generated. Asymmetric bispecific antibodies (BsAbs), which mostly consist of four distinct chains, are gaining prominence because of their capacity to concurrently target multiple antigens, thereby enhancing therapeutic outcomes.^[^ ^13^, ^14^, ^15^ ^]^ Although advancements like “knobs‐into‐holes”.^[^ ^16^ ^]^ and “Fc charge‐pair”,^[^ ^17^ ^]^ along with an orthogonal Fab interface,^[^ ^18^ ^]^ have enabled the intracellular assembly of BsAbs, the current single metabolic selection system falls short in selecting cells expressing multiple genes.

Because of the unique ability of inteins to self‐catalyze their excision from precursor proteins,^[^ ^19^ ^]^ split intein‐mediated protein ligation (SiMPl) has emerged as a potent synthetic biology tool.^[^ ^20^, ^21^ ^]^ The performance of trans‐splicing depends on the assembly rate of intein, the split site of the protein, and others.^[^ ^21^, ^22^, ^23^ ^]^ Intriguingly, the decreased performance of selection markers corresponds to enhanced selection stringency.^[^ ^24^ ^]^ By using the characteristics of SiMPl, we aimed to build a logic‐gated synthetic system to overcome the limitations of the traditional selection system by amplifying the selection stringency and expanding the selection cargo for next‐generation therapeutic proteins.

Herein, we have described a SiMPl‐GS selection system that operates on the AND‐gate principle, allowing simultaneous selection of multiple vectors using a single selection marker. Specifically, only cells containing all the required vectors survive. This system can be readily applied to the most widely used GS‐knockout host cell platform. We validated the rationally designed SiMPl‐GS system and demonstrated its application to various proteins of interest, such as fluorescent proteins, Fc‐fusion proteins, monoclonal antibodies (mAbs), and BsAbs. Additionally, we demonstrated multiple selections using orthogonal intein interfaces. This study illustrated the potential of SiMPl‐GS as a next‐generation therapeutic protein‐producing CLD.

## 2 Results and Discussion

### 2.1 Selection of Split Sites of GS Using a Computational Approach

Intein‐mediated trans‐splicing, which occurs in the cytoplasm.^[^ ^25^ ^]^ after a protein has already folded in the endoplasmic reticulum, is a critical step that can affect the functionality of the final protein. Therefore, it is essential to select an appropriate splitting site. To mitigate the potential functional loss of GS post‐trans‐splicing, we employed a computational approach to screen for the optimal split sites. This screening method considers three key factors: i) protein flexibility, ii) surface localization, and iii) evolutionary conservativeness.

**Figure 1:**
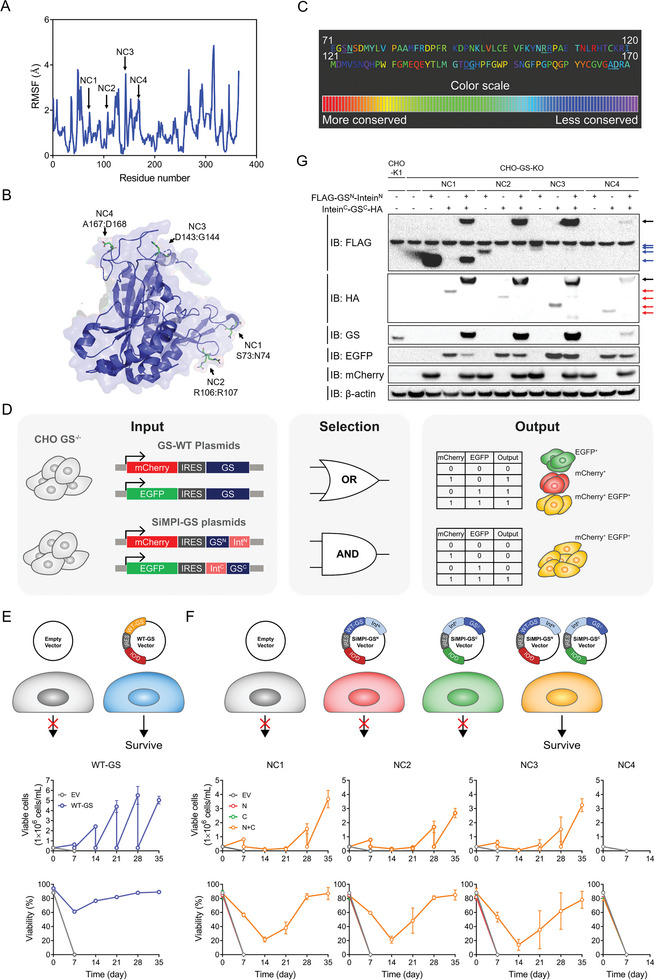
Rational design of split intein‐mediated protein ligation (SiMPl) of glutamine synthetase (GS). A) Root mean square fluctuation (RMSF) of Cα atoms from human glutamine synthetase (GS) (PDB: 2OJW) with potential split sites highlighted by black arrows. B) Crystal structure of human GS (PDB: 2OJW) with potential split sites highlighted by black arrows. Four potential split sites were referred to in the order of amino acid residue positions as NC1, NC2, NC3, and NC4. C) Evolutionary Trace analysis showing conservativeness of residues. Each amino acid was colored according to its evolutionary conservation, and potential split sites were underlined. D) Schematic representation of the logic‐gated selection system comparing conventional GS selection and SiMPl‐GS selection, using fluorescence reporter plasmids. E, F) Analysis of cell viability after transfection using specific plasmids in glutamine‐free medium (mean ± SD, n = 3). E) Viable cell concentration and F) viability during the selection of cells transfected with the indicated plasmids in the glutamine‐free medium. Three independent cell pools were generated. EV, transfection with pcDNA3.1 as an empty vector control; WT‐GS, transfection with WT‐GS encoding plasmid; N, transfection with SiMPl‐GS^N^ plasmid; C, transfection with SiMPl‐GS^C^ plasmid; N+C, co‐transfection with SiMPl‐GS^N^ and SiMPl‐GS^C^ plasmids. For N and C conditions, the total weight of transfected plasmids was adjusted with the pcDNA3.1 plasmid. Plasmid information is described in Table S1 (Supporting Information). G) Western blot analysis of split intein‐mediated trans‐splicing of GS. Whole cell lysates were subjected to western blotting with anti‐FLAG, HA, GS, EGFP, mCherry, and *β*‐actin antibodies. Data are presented as the means ± SDs.

Protein flexibility was assessed by analyzing the residue fluctuations (**Figure** **1**A). Additionally, split sites were strategically selected to ensure that they were surface‐exposed and not located in the active site (Figure 1B). Next, we investigated evolutionary conservativeness to identify less conserved residues throughout organisms, which indicated that these residues may not be critical for GS function (Figure 1C). Based on these criteria, we identified four potential cleavage sites: NC1, NC2, NC3, and NC4. As trans‐splicing resulted in a scar of six amino acid residues, the structure of these sites with the scar was further validated using AlphaFold2. No significant conformational changes at these sites were predicted using AlphaFold2 (Figure S1, Supporting Information). Finally, four split sites were selected as potential split sites for GS.

To evaluate the potential of the proposed split sites to enable cell survival in a glutamine‐free medium, we designed bicistronic expression vectors containing mCherry or enhanced green fluorescent protein (EGFP) linked to wild‐type GS (WT‐GS) or SiMPl‐GS split at various cleavage sites (NC1, NC2, NC3, and NC4) via the internal ribosome entry site (IRES) element (Figure 1D). The construction of SiMPl‐GS involved the fusion of the N‐terminal and C‐terminal domains of gp41‐1 intein to the corresponding domains of GS.^[^ ^20^ ^]^ The pcDNA3.1 plasmid was used as an empty vector (EV) control. GS‐knockout (CHO‐GS KO) cells were transfected with bicistronic expression vectors or EV. As expected, cells co‐transfected with mCherry and EGFP plasmids expressing WT‐GS survived in glutamine‐free media (Figure 1E). In contrast, cells transfected with the SiMPl‐GS^N^ plasmid alone or the SiMPl‐GS^C^ plasmid alone did not survive in glutamine‐free media (Figure 1E). However, cells transfected with both SiMPl‐GS^N^ and SiMPl‐GS^C^ plasmids, except for the GS split at NC4, survived in the glutamine‐free medium, suggesting that NC1, NC2, and NC3 are appropriate split sites for GS (Figure 1F).

To confirm in vitro assembly of SiMPl‐GS at all four candidate splitting sites, SiMPl‐GS^N^ and SiMPl‐GS^C^ were tagged with FLAG and HA, respectively. Subsequently, the cells were transfected with the indicated plasmids, and after 2 days of cultivation, cell lysates were prepared and subjected to western blot analysis (Figure 1G). Regardless of the splitting sites, the expression levels of mCherry and EGFP were similar (Figure 1G; Figure S2, Supporting Information). However, unlike SiMPl‐GS at the NC1, NC2, and NC3 sites, SiMPl‐GS at NC4 was marginally assembled, which was significantly lower than that of the endogenous GS in CHO‐K1 cells. When the three SiMPl‐GS variants (NC1, NC2, and NC3) and WT‐GS cell pools were cultivated in glutamine‐free selection medium, glutamine was detected at 0.10–0.17 mm in the glutamine‐free medium after 3 days of cultivation, suggesting that they all synthesized glutamine (Figure S3, Supporting Information). Therefore, we further validated the three selected split sites (NC1, NC2, and NC3).

### 2.2 Efficient Selection and Enrichment of Cells Highly Expressing Reporter Genes by SiMPl‐GS System

**Figure 2:**
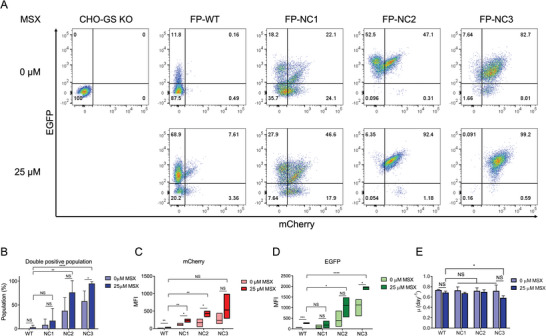
Selection efficiency of the SiMPl‐GS system for reporter‐expressing cell pools. A) Representative flow cytometry analysis of fluorescence proteins (FP)‐expressing cell pools generated with WT‐GS or SiMPl‐GS selection system. Once the cell pools were generated, cells were further selected using 25 µm methionine sulfoximine (MSX). All samples were analyzed when their viability reached above 90%. B–D) Fluorescence double‐positive populations (B) and mean fluorescence intensity (MFI) of mCherry (C) and EGFP (D) in the indicated cells (mean ± SD, *n* = 3). E) The specific growth rate (µ) of the FP cells was determined based on viable cell concentrations from day 0 to day 3 (mean ± SD, *n* = 3). Data are presented as the means ± SDs. *p*‐values were calculated by paired two‐tailed Student's *t*‐test, NS, not significant; *p < 0.05; **p < 0.01; ***p < 0.001; ****p < 0.0001.

To evaluate the selection efficacy of the novel SiMPl‐GS system, the three SiMPl‐GS variants and WT‐GS cell pools were analyzed using flow cytometry. When cell viability was recovered, the double‐positive cell population expressing both mCherry and EGFP in the WT‐GS cell pool was 0.9% ± 0.1% (*n* = 3). In contrast, the double positive cell populations in the three SiMPl‐GS variant cell pools (NC1, NC2, and NC3) were 21.0% ± 19.5%, 36.5% ± 0.7%, and 76.0% ± 7.5%, respectively, which was 23–84 times higher than that in the WT‐GS cell pool (**Figure** **2**A,B). Likewise, the expression levels of mCherry and EGFP in the three SiMPl‐GS variant cell pools were significantly higher than those in the WT‐GS cell pool. The mean fluorescence intensities (MFIs) of mCherry and EGFP in the WT‐GS cell pool were 22.6 ± 4.2 and 93.9 ± 5.8, respectively. Those of NC1, NC2, and NC3 were (219.7 ± 112.9 and 302.7 ± 91.6), (194.1 ± 90.9 and 317.3 ± 118.8), and (519.3 ± 222.4 and 1082.3 ± 296.5), respectively (Figure 2C,D). The differences in selection efficiency and expression levels observed among NC1, NC2, and NC3 SiMPl‐GS variants may be attributed to structural variations introduced by trans‐splicing, which results in a product that contains a scar of six residues.^[^ ^20^ ^]^ These structural differences can affect protein function, leading to varying selection efficiency and expression levels among the variants.

The selection efficiency and expression levels of mCherry and EGFP in the three SiMPl‐GS variant cell pools that survived glutamine deficiency were further improved by treatment with methionine sulfoximine (MSX), a GS inhibitor (Figure 2A,B). When treated with 25 µm MSX, the double positive cell populations in the three SiMPl‐GS variant cell pools (NC1, NC2, and NC3) were 17.1% ± 25.6%, 76.4% ± 24.7%, and 95.2% ± 3.6%, respectively. Likewise, treatment with 25 µm MSX increased the expression levels of mCherry and EGFP by 2–4 folds in the three SiMPl‐GS variant cell pools (NC1, NC2, and NC3) (Figure 2C,D).

When developing new selection markers, it is essential to ensure that these markers do not have detrimental effects on cell growth. The specific growth rate (µ) of the three SiMPl‐GS variant cell pools was similar to that of the WT‐GS cell pool (Figure 2E). In addition, treatment with 25 µm MSX did not decrease µ of the three SiMPl‐GS variant cell pools significantly. Only the NC3 cell pool treated with 25 µm MSX showed reduced µ, compared with the WT‐GS cell pool (Figure 2E). The NC3 variant exhibited the highest selection stringency, likely due to the greater burden imposed by MSX on glutamine production. As glutamine regulates cell proliferation,^[^ ^26^, ^27^ ^]^ increased sensitivity of the NC3 variant to MSX could result in a reduced growth rate. Taken together, SiMPl‐GS shows potential as a powerful selection system for enriching cells that highly express the genes of interest (GOI).

### 2.3 SiMPl‐GS System for Fc‐Fusion Protein Production

**Figure 3:**
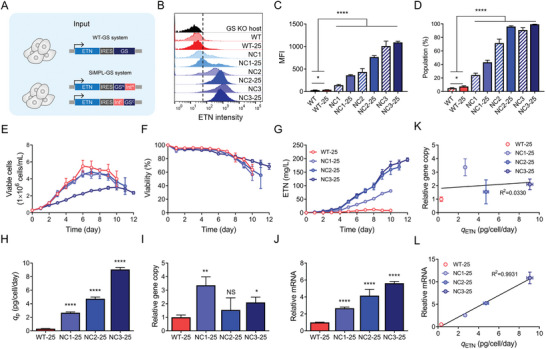
SiMPl‐GS system for generating Fc‐fusion protein‐producing cells. A) Schematic of Fc‐fusion protein, etanercept (ETN)‐producing WT‐GS (top) and SiMPl‐GS (bottom) plasmids. B–D) Flow cytometry analysis of ETN‐producing cell pools (mean ± SD, *n* = 3). Cell pools were generated by transfecting ETN‐expressing plasmids. Cells were further incubated with 25 µm MSX. B) Histogram, C) MFI, and D) ETN‐producing population were determined by staining ETN on the cell surface. The vertical dashed line indicates the threshold set based on the CHO‐GS KO host cells. E–G) Profiles of cell growth (E), viability (F), and ETN concentration (G) of ETN‐producing cell pools during culture (mean ± SD, n = 3). H) Specific productivity (qETN) derived from (E) and (G) (mean ± SD, n  =  3). I) Relative gene copy number of ETN normalized to C1GALT1C1 (mean ± SD, n = 3). J) Relative mRNA expression level of ETN normalized to GAPDH (mean ± SD, n = 3). K) Correlation analysis between qETN and relative mRNA expression levels of cell pools (mean ± SD, n  =  3). L) Correlation analysis between qETN and relative gene copy number of cell pools (mean ± SD, n = 3). Data are presented as the means ± SDs. The coefficient of determination (R2) was calculated using linear regression. p‐values were calculated by paired two‐tailed Student's t‐test, NS, not significant; *p < 0.05; **p < 0.01; ****p < 0.0001.

To evaluate the potential of the SiMPl‐GS system for the development of therapeutic protein‐producing cells, we constructed etanercept (ETN), an Fc‐fusion protein‐expressing WT‐GS, and SiMPl‐GS plasmids (**Figure** **3**A). Subsequently, three SiMPl‐GS variants and WT‐GS cell pools producing ETN (ETN‐NC1, ETN‐NC2, ETN‐NC3, and ETN‐WT‐GS) were generated by transfecting CHO‐GS KO cells with the same amounts of the corresponding plasmids. To simplify the CLD process, we bypassed the traditional minipool selection step, which is typically performed after transfection. WT‐GS and SiMPl‐GS cell pools were selected until cell viability surpassed 90% (Figure S4, Supporting Information). Selected cell pools were analyzed using flow cytometry to estimate the cell surface ETN of individual cells in the cell pool, which correlated with specific ETN productivity (*q*_ETN_).

Consistent with the cell pools expressing mCherry and EGFP (Figure 2), the SiMPl‐GS variant cell pools producing ETN had higher ETN expression levels than ETN‐WT‐GS (Figure 3B). Among the three SiMPl‐GS variant cell pools, ETN‐NC3 showed the highest cell surface ETN expression levels. The ETN‐producing population and MFI of ETN‐N3 were 90.9% ± 3.6% and 1010.0 ± 110.3, respectively, whereas those of ETN‐WT‐GS were 4.9% ± 0.9% and 31.2% ± 3.4, respectively (Figure 3C,D). Further selection with 25 µm MSX increased the ETN‐producing population of ETN‐N3 to 99.4% ± 0.2% but did not significantly increase the expression of cell surface ETN. This was probably because the population of ETN‐producing cells was already high. The MSX selection system eliminates non‐ or low‐producers and does not improve *q* of individual cells.^[^ ^10^ ^]^ In contrast, for ETN‐NC1 and ETN‐NC2, both the ETN‐producing populations and the expression levels of cell surface ETN were significantly increased at 25 µm MSX (Figure 3C,D).

To further characterize the SiMPl‐GS variant and WT‐GS cell pools, cells were seeded at 3 × 10^5^ cells mL^−1^ in Erlenmeyer flasks containing 30 mL of glutamine‐free medium containing 25 µm MSX. Among the three SiMPl‐GS variant cell pools, only ETN‐NC3 showed significant growth suppression compared with ETN‐WT‐GS at 25 µm MSX (Figure 3E,F). However, despite growth suppression, the ETN concentration in ETN‐NC3 was much higher than that in ETN‐WT‐GS owing to the increased *q*_ETN_ and ETN‐producing populations (Figure 3G). In the absence of MSX, ETN‐NC3 grew well while maintaining a high *q*_ETN_ (Figure S5, Supporting Information). In the presence of 25 µm MSX, *q*_ETN_ of ETN‐NC1, ETN‐NC2, and ETN‐NC3 cell pools were 8.4‐, 14.9‐, and 28.6‐fold higher than that of ETN‐WT‐GS, respectively (Figure 3H).

ETN gene copy number and mRNA expression levels in the SiMPl‐GS variant cell pools were higher than those in the WT‐GS cell pools (Figure 3I,J). Theoretically, the genomic DNA (gDNA) copy number of the SiMPl‐GS system is expected to be approximately twice that of the WT‐GS system because the SiMPl‐GS system requires the integration of both SiMPl‐GS^N^ and SiMPl‐GS^C^ into the host genome. Interestingly, *q*_ETN_ of the SiMPl‐GS variant cell pools did not correlate with the ETN gene copy number (R^2^ = 0.0330) (Figure 3K). However, *q*_ETN_ highly correlated with mRNA expression levels (R^2^ = 0.9931), suggesting that selection stringency mainly depended on mRNA and protein expression levels (Figure 3L). Collectively, SiMPl‐GS outperformed WT‐GS in establishing cell pools that produce therapeutic Fc‐fusion proteins. In addition, the selection stringency of SiMPl‐GS depended on the split sites of the GS.

### 2.4 Bicistronic SiMPl‐GS System for mAb Production

We designed bicistronic plasmids to generate mAb‐producing cell pools. Unlike single‐chain Fc‐fusion proteins, mAbs are composed of two distinct chains: a heavy chain (HC) and a light chain (LC). Given the presence of a degron sequence in the N‐terminal region of GS.^[^ ^28^ ^]^ that can induce proteasomal degradation, we explored different configurations of LC and HC expression. For efficient mAb production by CHO cells, the ratio of HC to LC expression is optimized, and LC genes are often expressed at higher levels than HC genes.^[^ ^29^ ^]^ Therefore, we hypothesized that LC expression using SiMPl‐GS^N^ and HC expression using SiMPl‐GS^C^ (LC:GS^N^‐HC:GS^C^) would be more efficient for mAb production than the reverse configuration (HC:GS^N^‐LC:GS^C^).

**Figure 4:**
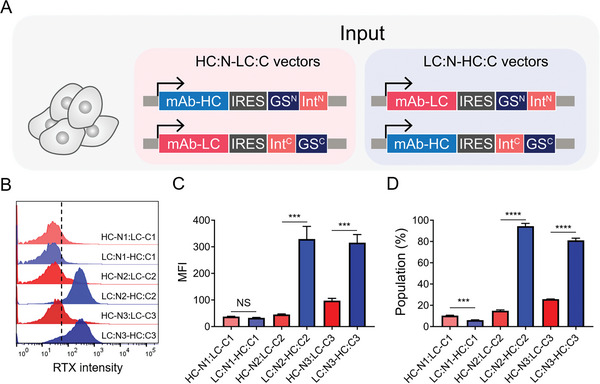
Bicistronic SiMPl‐GS dual‐selection system for generating monoclonal antibody (mAb)‐producing cells. A) Schematic of bicistronically linked mAb‐producing plasmids. Two configurations were designed: one with the HC linked to SiMPl‐GS^N^ and the LC to SiMPl‐GS^C^ (HC:GS^N^‐LC:GS^C^) (left) and the other with the LC linked to SiMPl‐GS^N^ and the HC to SiMPl‐GS^C^ (LC:GS^N^‐HC:GS^C^) (right). Both constructs were developed using three variants of SiMPl‐GS. B–D) Flow cytometric analysis of bicistronic mAb‐producing cell pools (mean ± SD, n = 3). B) Histogram, C) MFI, and bicistronic SiMPl‐GS, mAb‐producing population D) determined by staining mAb on the cell surface. The vertical dashed line indicates the threshold set based on the CHO‐GS KO host cells. Data are presented as the means ± SDs. p‐values were calculated by paired two‐tailed Student's *t*‐test, NS, not significant; ***p < 0.001; ****p < 0.0001.

To test this, we constructed two different mAb‐expressing dual plasmids, each linked bicistronically to SiMPl‐GS variants (**Figure** **4**A). Among the cleavage sites, NC1 showed suboptimal efficiency for selecting mAb‐expressing cell pools (Figure 4B). Flow cytometry analysis showed that for NC2 and NC3, the LC:GS^N^‐HC:GS^C^ configuration yielded higher mAb production than the HC:GS^N^‐LC:GS^C^ configuration (Figure 4B,C). The mAb‐producing populations of HC:GS^N^‐LC:GS^C^ configurations were 14.9% ± 0.8% for NC2 and 25.8% ± 0.2% for NC3, whereas the LC:GS^N^‐HC:GS^C^ configurations were 94.4% ± 2.7% for NC2 and 81.0% ± 2.1% for NC3 (Figure 4D). Thus, we confirmed that higher levels of LC expression were more favorable than HC expression for mAb production in the bicistronically linked SiMPl‐GS dual‐selection system.

### 2.5 SiMPl‐GS System in Independent Promoter‐Driven mAb Expression Vectors

To evaluate the selection efficiency of the SiMPl‐GS system in conventional three‐promoter‐driven mAb‐expressing plasmids, we generated rituximab‐producing cell pools, referred to as mAb‐WT, mAb‐NC1, mAb‐NC2, and mAb‐NC3, using the conventional three promoter‐driven mAb‐expressing plasmids as the backbone (Figure S6A, Supporting Information).^[^ ^30^ ^]^ When the expression of LC, GS, and HC is regulated by distinct promoters, this raises challenges for conventional random integration methods that involve the insertion of random segments of circular plasmids into random locations in the host genome. As a result, selection is biased toward cells expressing GS, regardless of the expression of HC or LC, resulting in less efficient selection. In the mAb‐WT, mAb‐NC1, and mAb‐NC2 cell pools, mAb‐producing cells were rarely detected and were not increased by treatment with 25 µm MSX (Figure S6B–D, Supporting Information). In contrast, the mAb‐producing population of the mAb‐NC3 cell pool, with NC3 showing the highest selection stringency, was 73.0% ± 4.2% and further increased to 89.8% ± 1.4% by treatment with 25 µm MSX (Figure S6D, Supporting Information). When cultured in Erlenmeyer flasks, the mAb‐NC3 cell pool had the highest mAb concentration of 362.7 ± 9.1 mg L^−1^ (Figure S6E–G, Supporting Information). Analysis of mRNA expression levels revealed that only the mAb‐NC3 cell pool showed significant expression levels in both LC and HC (Figure S6H, Supporting Information). These results collectively affirm the potential of SiMPl‐GS with an appropriate split site as a powerful selection system.

**Figure 5:**
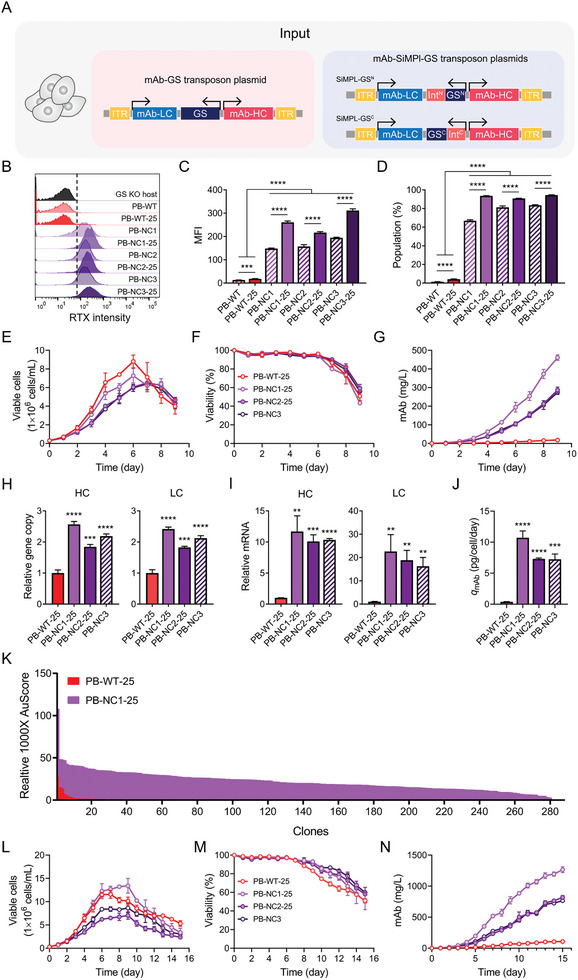
SiMPl‐GS system for generating transposon‐based mAb‐producing cells. A) Schematic of PiggyBac (PB) transposon‐based mAb‐producing WT‐GS (left) and SiMPl‐GS (right) plasmids. ITR, inverted terminal repeat. B–D) Flow cytometric analysis of mAb‐producing cell pools generated using PB transposon (mean ± SD, *n* = 3). Cells were further incubated with 25 µm MSX. B) Histogram, C) MFI, and D) mAb‐producing population determined by staining mAb on the cell surface. The vertical dashed line indicates the threshold set based on the CHO‐GS KO host cells. E–G) Profiles of cell growth (E), viability (F), and mAb concentration (G) of mAb‐producing cell pools during culture (mean ± SD, *n* = 3). H) Relative gene copy number of HC and LC normalized to *C1GALT1C1* (mean ± SD, *n*  =  3). I) Relative mRNA expression level of HC and LC normalized to *GAPDH* (mean ± SD, *n* = 3). J) *q*_mAb_ derived from (E) and (G) (mean ± SD, *n* = 3). K) Clonal productivity assessment of PB‐WT‐25 and PB‐NC1‐25 cell pools using the Beacon optofluidic system. Secretion assay scores (AU scores) of each pen loaded with a single cell were measured on day 3. The analysis was conducted on 280 single cells randomly selected from each cell pool. L–N) L) Profiles of cell growth, M) viability, and N) mAb concentration of mAb‐producing cell pools during fed‐batch culture (mean ± SD, *n* = 3). Data are presented as the means ± SDs. *p*‐values were calculated by paired two‐tailed Student's *t*‐test, NS, not significant; **p < 0.01; ***p < 0.001; ****p < 0.0001.

Next, we investigated the compatibility of the SiMPl‐GS selection system with transposon‐mediated gene transfer. Transposon systems such as PiggyBac (PB) are genetic elements that integrate gene fragments of inverted terminal repeat (ITR) sequences into chromosomes.^[^ ^31^ ^]^ Transposon systems are known for their ability to enhance stable CLD efficiency.^[^ ^32^ ^]^ Consequently, we incorporated ITRs at both ends of the mAb expression vectors, which were used to generate mAb‐producing cell pools referred to as mAb‐PB‐WT, mAb‐PB‐NC1, mAb‐PB‐NC2, and mAb‐PB‐NC3 (**Figure** **5**A). Initially, we confirmed that the PB‐mediated gene transfer improved the efficiency of the mAb‐producing cell pool (Figure S7, Supporting Information).

All mAb‐expressing cell pools generated with the PB system, especially mAb‐PB‐NC1 and mAb‐PB‐NC2, exhibited enhanced mAb expression compared with those generated without the PB system (Figure S8A–C, Supporting Information). The mAb‐producing populations of the mAb‐PB‐NC1 and mAb‐PB‐NC2 were 55.5% ± 0.8% and 81.7% ± 0.9%, respectively, whereas those of the mAb‐NC1 and mAb‐NC2 were 0.4% ± 0.1% and 0.3% ± 0.1%, respectively. When cultured with 25 µm of MSX, all PB‐based mAb‐expressing cell pools showed increased mAb‐producing populations and mAb expression levels (Figure 5B–D). However, like ETN‐NC3, only mAb‐PB‐NC3 showed significant growth suppression at 25 µm MSX. Taken together, the SiMPl‐GS system can be used in conjunction with other integration methods, such as the PB system, to improve stable CLD efficiency.

To further characterize PB‐based mAb‐expressing cell pools selected at 25 µm MSX (mAb‐PB‐WT‐25, mAb‐PB‐NC1‐25, and mAb‐PB‐NC2‐25) cell pools were cultivated at 25 µm MSX. The mAb‐PB‐NC3 cell pool was cultured without MSX. Among these, mAb‐PB‐WT‐25 showed the highest cell growth but the lowest mAb production (Figure 5E–G). The mAb‐PB‐NC1‐25 showed the highest mAb concentration of 461.0 ± 14.8 mg L^−1^, which was 25.2‐fold higher than that of mAb‐PB‐WT‐25.

Similar to the ETN‐producing cell pools (Figure 3), HC and LC gene copy numbers almost doubled in the SiMPI‐GS system with PB (Figure 5H). Furthermore, the mRNA expression levels of HC and LC and *q*_mAb_ increased more than ten‐fold in the SiMPl‐GS selection system with PB (Figure 5I,J). Thus, we confirmed the compatibility of the SiMPl‐GS selection system with the PB.

To further investigate the mAb productivity of individual clones within a cell pool, we randomly isolated 280 single cells from each cell pool (mAb‐PB‐WT‐25 and mAb‐PB‐NC1) using the Beacon optofluidic system and measured the secretion assay score (AU score), which reflects the amount of mAb secreted (Figure 5K). For PB‐WT‐25, only 77 of the 280 clones produced mAbs, with most showing negligible AU scores. In contrast, for mAb‐PB‐NC1‐25, all 280 clones were found to produce mAbs at significantly higher levels than the mAb‐PB‐WT‐25‐derived clones.

To evaluate the potential of the SiMPl‐GS selection system for the rapid production of significant quantities of mAbs, we cultivated PB‐based mAb‐expressing cell pools in fed‐batch culture, which is the most widely used method for large‐scale mAb production (Figure 5L–N). The final mAb concentrations for mAb‐PB‐WT‐25, mAb‐PB‐NC1‐25, mAb‐PB‐NC2‐25, and mAb‐PB‐NC3 were 108.7 ± 5.5, 1264.3 ± 46.7, 869.7 ± 21.5, and 767 ± 22.6 mg L^−1^, respectively, suggesting that the SiMPl‐GS selection system can be effectively employed for rapidly yielding substantial quantities of mAbs at the cell pool stage. Collectively, these findings highlight the efficacy of the SiMPl‐GS selection system for mAb‐producing CLD.

### 2.6 Expanded SiMPl‐GS Quad‐Selection System for BsAb Production

**Figure 6:**
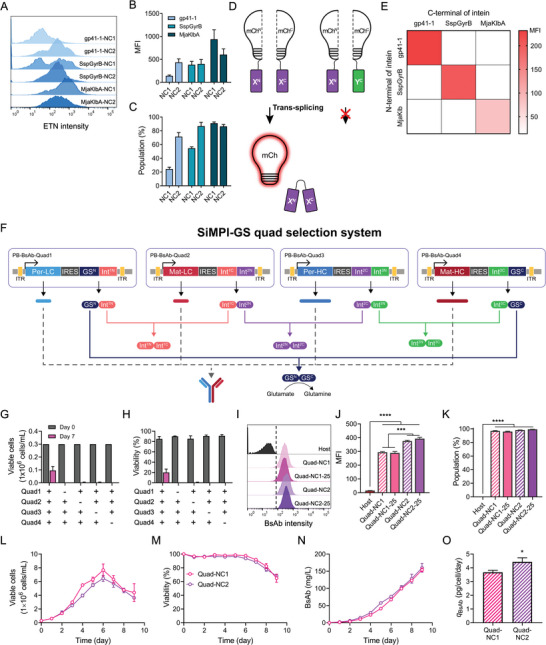
Expanded logic‐gated SiMPl‐GS system for multiple selections. A–C) Flow cytometric analysis of active split inteins (gp41‐1, SspGyrB, and MjaKlbA) for generating ETN‐producing cell pools (mean ± SD, *n* = 3). A) Histogram, B) MFI, and C) ETN‐producing population determined by staining ETN on the cell surface. Split sites referred to as NC1 and NC2 of GS were used. D) Construct of SiMPl‐mcherry (mCh)‐expressing plasmids with split inteins. X and Y are split inteins. E) Characterization of orthogonality matrix of split intein pairs (*n* = 3). Color gradients represent the MFI of trans‐spliced mCherry measured 2 days post‐transfection. F) Schematic illustration representing three split intein‐integrated SiMPl‐GS Quad‐selection systems for selecting multiple genes. Three split inteins (gp41‐1, SspGyrB, and MjaKlbA) were used to construct a Quad‐selection system. NC1 and NC2 of GS were used. Bispecific antibodies (BsAb) consisting of HC and LC of pertuzumab (Per) and matuzumab (Mat) were used as genes of interest. G, H) Viable cells (G) and viability (H) of cells transfected with the indicated plasmids were measured on days 3 and 7 (mean ± SD, *n*  =  3). Three independent cell pools were generated. I–K) Flow cytometric analysis of BsAb‐producing cell pools generated using PB transposon (mean ± SD, *n* = 3). Histogram (I), MFI (J), and BsAb‐producing population (K) determined by staining BsAb on the cell surface. The vertical dashed line indicates the threshold set based on the CHO‐GS KO host cells. L–N) Profiles of cell growth (L), viability (M), and mAb concentration (N) of BsAb‐producing cell pools during culture (mean ± SD, *n* = 3). O) *q*_BsAb_ derived from L, N) (mean ± SD, *n* = 3). Data are presented as the means ± SDs. *p*‐values were calculated by paired two‐tailed Student's *t*‐test, *p < 0.05; ***p < 0.001; ****p < 0.0001.

To generate cell pools that produce more complex proteins, such as BsAbs and tri‐specific antibodies, we explored the expandability of the SiMPl‐GS system. This system called the SiMPl‐GS Quad‐selection system, was designed to mimic four selection markers using only the GS selection system. First, additional split inteins (NpuDnaE, Cth‐Ter, SspGyrB, and MjaKlbA), whose efficient trans‐splicing capabilities have previously been demonstrated in prokaryotic cells,^[^ ^20^ ^]^ were selected for compatibility testing of GS trans‐splicing. ETN‐expressing plasmids were constructed using split inteins. Cell pools with different intein‐mediated SiMPl‐GSs were generated and ETN‐producing cell pools were analyzed using flow cytometry. Among the inteins evaluated, only the cells transfected with SspGyrB or MjaKlbA inteins encoding SiMPl‐GS were effectively recovered, demonstrating compatibility with SiMPl‐GS selection (**Figure** **6**A–C). Next, to evaluate the orthogonality of the split inteins, we used the previously developed split mCherry platform to construct SiMPl‐mCherry expression plasmids incorporating various split inteins, a total of six plasmids (Figure 6D). For evaluation, cells were co‐transfected with two vectors, each containing one N‐terminal domain or one C‐terminal domain, selected from the six plasmids. 2 days post‐transfection, mCherry expression in transfected cell pools was assessed using flow cytometry. Of the nine different cases, mCherry expression was observed only in cells transfected with two vectors, each containing an N‐terminal‐ or C‐terminal domain from the same split intein, suggesting that cross‐interactions between the different split intein pairs were negligible (Figure 6E).

We designed a SiMPl‐GS Quad‐selection system incorporating NC1 and NC2 variants to facilitate the generation of cells expressing matuzumab‐pertuzumab BsAb (Figure 6F). The core mechanism involves multiple orthogonally split intein interfaces that facilitate several rounds of trans‐splicing, eventually generating an assembled GS. Four plasmids, denoted as Quad1, Quad2, Quad3, and Quad4, were constructed to initiate trans‐splicing through the interaction of three pairs of split intein domains. The Quad1 and Quad4 plasmids contain N‐terminal‐ and C‐terminal GS domains, respectively. This configuration is intended to drive several rounds of trans‐splicing, ultimately resulting in the assembly of a functional GS. Each of the four plasmids was bicistronically linked to one of the four chains of matuzumab‐pertuzumab BsAb along with a selection marker. This design ensured that only cells that correctly integrated and expressed all parts of the Quad system survived the selection process, thereby facilitating the identification of productive BsAb‐expressing cell lines.

To determine whether the SiMPl‐GS Quad‐selection system was fully operational only when all four vectors were present, the cells were transfected with all four SiMPl‐GS Quad‐NC1 plasmids or with a subset of three plasmids. 2 days after transfection, the transfected cells were cultured in a glutamine‐free medium for 7 days. Only cells transfected with all four vectors survived and were named Quad‐NC1, confirming the efficacy of the novel SiMPl‐GS Quad‐selection system operated by single GS selection (Figure 6G,H).

To confirm the in vitro assembly of SiMPl‐GS at different splitting sites, we generated BsAb‐expressing cell pools using SiMPl‐GS Quad NC2 plasmids (Quad‐NC2). WT‐GS and SiMPl‐GS pools were selected until cell viability surpassed 90% (Figure S9, Supporting Information). Regardless of the split site, almost all cells that survived the selection process produced BsAbs, which were measured using flow cytometry (Figure 6I–K). The BsAb‐producing populations of the Quad‐NC1 and Quad‐NC2 were 96.8% ± 0.8% and 98.3% ± 0.2%, respectively. Therefore, further selection at 25 µm MSX was unnecessary.

In addition, the MFIs of BsAb of Quad‐NC1 and Quad‐NC2 were 294.3 ± 4.9 and 375.7 ± 5.7, respectively, suggesting that *q*_BsAb_ of Quad‐NC2 was higher than that of Quad‐NC1. To confirm this, Quad‐NC1 and Quad‐NC2 cell pools were cultured in Erlenmeyer flasks. Quad‐NC1 grew better than Quad‐NC2, but their final BsAb concentrations were similar, confirming that the *q*_BsAb_ of Quad‐NC2 (3.67 ± 0.13 pg cell^−1^ day^−1^) was higher than that of Quad‐NC1 (4.43 ± 0.13 pg cell^−1^ day^−1^) (Figure 6L–O). The final BsAb concentrations of Quad‐NC1 and Quad‐NC2 were 154.0 ± 5.6 and 160.0 ± 12.8 mg L^−1^, respectively. Hence, the expanded SiMPl‐GS selection system is a promising strategy for generating cell pools expressing multiple genes.

## 3 Conclusion

Mammalian expression platforms for the production of therapeutic proteins are becoming increasingly important as more diverse and complex protein drugs are under development. Moreover, in public health emergencies, such as the COVID‐19 pandemic, sufficient amounts of candidate proteins must be produced at the cell pool stage for rapid drug discovery.^[^ ^5^ ^]^ However, current GS‐based selection systems, which are widely used to obtain high‐producing clones, are often inefficient and time‐consuming.

Attenuating GS using mutations or weak promoters improves selection efficiency; however, the expandability of attenuated GS for selecting multiple genes remains similar to that of WT‐GS.^[^ ^24^, ^33^ ^]^ To select multiple genes, various selection markers, such as proline,^[^ ^34^, ^35^ ^]^ asparagine,^[^ ^36^ ^]^ pyrimidine, and purine,^[^ ^37^ ^]^ have been explored. However, this has the disadvantage of requiring the creation of new knockout hosts and validation of the expression stability and quality attributes of the product. Taking advantage of the auxotrophic GS selection machinery, the SiMPl‐GS selection system can be readily applied to widely used GS‐knockout host cells.^[^ ^38^ ^]^

We developed an AND‐gate SiMPl‐GS selection system by fusing the N‐terminal‐ and C‐terminal GS with split inteins. Using a computational design‐based approach for selection and experimental validation, we identified the three most promising SiMPl‐GS split sites. The SiMPl‐GS selection system streamlined the CLD process to enrich high‐producing cells by depleting low‐/non‐producing cells. The efficacy of the SiMPl‐GS selection system was demonstrated using various types of proteins of interest, such as Fc‐fusion proteins, mAbs, and BsAbs. Regardless of the protein of interest, SiMPl‐GS consistently outperformed WT‐GS in enriching producing cells in terms of producer cell population and product expression levels. Even without MSX, the selection stringency of the SiMPl‐GS system was superior to that of the WT‐GS system using MSX. However, the higher selection stringency of the SiMPl‐GS system resulted in a slower recovery rate than that of the WT‐GS system. The recovery of producer cells can be accelerated by optimizing growth media and supplements, as previously observed.^[^ ^38^ ^]^

The SiMPl‐GS selection system provides tunable selection stringency by modulating several components. Selection stringency can be modulated by selecting the split site on the protein of interest.^[^ ^22^, ^23^ ^]^ Among the three split sites in GS, NC1, and NC3 had the lowest and highest selection stringencies, respectively. These variations in selection stringency highlight the importance of split‐site selection for modulating selection selectivity. The use of inteins introduces another layer of tunability. When evaluating five inteins, including gp41‐1 with a high trans‐splicing rate and NpuDnaE with a low trans‐splicing rate, we observed a broad spectrum of cell survival outcomes during pool selection.^[^ ^22^, ^39^ ^]^ In particular, the use of NpuDnaE resulted in complete cell death during selection, likely because of excessively high selection stringency. This finding demonstrates that intein selection requires a delicate balance because both the trans‐splicing rate and nature of the intein can greatly affect the overall efficiency and stringency of the system.

Recently, tools for integration into the host genome, including transposon‐based integration, have been developed and are increasingly used for CLD.^[^ ^32^ ^]^ We also investigated the compatibility of the SiMPl‐GS system with transposon‐based integration. When cooperating with transposons, the SiMPl‐GS system significantly increased the selection efficiency (Figure 4B–D). These results suggest that the efficiency of the SiMPl‐GS selection system can be extended to other gene integration methods, such as recombinase‐mediated cassette exchange (RMCE) and integrase‐based methods.^[^ ^40^ ^]^ In particular, RMCE often involves replacing the fluorescent protein‐coding gene cassette with a GOI cassette and subsequently sorting the fluorescence‐negative population. However, using dual landing pad RMCE, this fluorescence‐negative population was only 0.01–2%.^[^ ^41^, ^42^ ^]^ Therefore, antibiotic selection is necessary to enrich the fluorescence‐negative population.^[^ ^42^ ^]^ The SiMPl‐GS system can directly enrich populations, excluding the need for antibiotic use, particularly in cases where RMCE or multiple landing pads have occurred. Therefore, the SiMPl‐GS system can effectively enrich high‐producing cells across a wide range of integration strategies, making it a versatile tool for optimizing CLD.

Another advantage of using the AND‐gate SiMPl‐GS selection system is its ability to select multiple GOI‐expressing cells. The development of Fab interfaces.^[^ ^18^ ^]^ has enabled the expression of correctly assembled BsAbs directly within cells, eliminating the need for a cumbersome Fab‐arm exchange process.^[^ ^43^ ^]^ traditionally required to assemble two separately expressed mAbs. However, markers are still required to select multigene‐integrated cells. To generate a cell line that produces three‐chain bispecific scFv‐Fab‐Fc, three selection markers are required.^[^ ^43^ ^]^ In contrast, our Quad‐selection approach simplifies CLD for complex antibody formats by requiring only a single selection marker.

We demonstrated the feasibility of a Quad‐selection system that achieved a production population of more than 96%. The utility of multi‐selection is not limited to GS but can also be extended to other proteins, such as antibiotic‐resistant genes, reporter genes, and transcription factors.^[^ ^20^, ^22^ ^]^ Recent synthetic biology research has typically required the integration and expression of multiple genes for precise and complex interactive circuit design.^[^ ^44^, ^45^, ^46^ ^]^ Although we have not yet optimized this system using other cell types and proteins, the multiple‐selection system developed herein is likely to be widely used to select cells that integrate multiple components.

In this study, we demonstrated that the SiMPl‐GS selection system is superior to the WT‐GS system. However, the productivity of these systems is not typical from an industrial perspective because standard GS vectors were used. Therefore, for industrial applications, the productivity of the SiMPl‐GS selection system must be further improved by optimizing the integration method, using a strong promoter, or incorporating UCOE elements.

**Figure 7:**
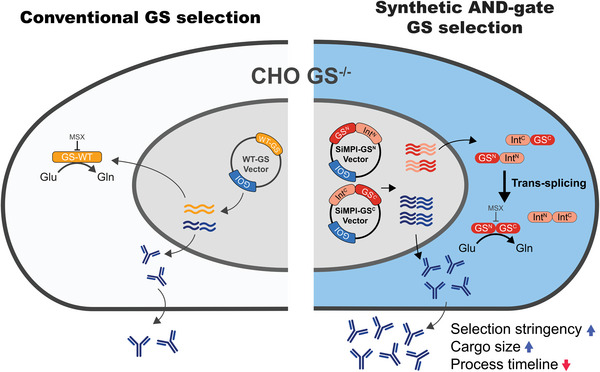
Schematic illustration of AND‐gate SiMPl‐ GS selection system. Comparison of conventional GS selection (left) and SiMPl‐GS selection (right) systems. CHO cells deficient in GS are transfected separately with WT‐GS or co‐transfected with SiMPl‐GS^N^ and SiMPl‐GS^C^ plasmids that carry the genes of interest. In the SiMPl‐GS selection system, the N‐terminal and C‐terminal domains of GS are fused to the N‐terminal and C‐terminal fragments of the split intein, respectively. These domains are expressed independently within GS‐knockout cells and subsequently undergo trans‐splicing within the cytoplasm and reconstitution of a functional GS enzyme. The reconstituted GS can synthesize glutamine, thus allowing the cells to survive in a glutamine‐free selection medium.

In summary, the AND‐gate synthetic SiMPl‐GS selection system presented in this study is a powerful toolbox for streamlining the CLD process. As summarized in **Figure** **7**, the SiMPl‐GS system can be directly adopted in widely used GS‐knockout host cells by exploiting the auxotrophic GS selection machinery. In addition, multiple SiMPl interfaces have been successfully applied for multiple‐plasmid selection. Taken together, SiMPl‐GS and multiple Quad selections have a significant potential for generating cell lines that produce therapeutic proteins, including BsAbs.

## 4 Experimental Section

### Computational Design

The rational design of SiMPl‐GS was based on the crystal structure of GS (PDB: 2OJW). Computational tools, including the CABS‐flex standalone package,^[^ ^47^ ^]^ PyMOL (v 2.5.4), and Evolutionary Trace,^[^ ^48^ ^]^ were employed for analysis and visualization. Predictive modeling of the structural changes at each SiMPl‐GS split site was conducted using AlphaFold2 Colab.^[^ ^49^ ^]^ Subsequent alignment of the predicted structures was performed using RaptorX.^[^ ^50^ ^]^ and visualized using PyMOL.

### Cell Lines, Culture Media, and Culture Maintenance

CHO‐K1 and CHO‐GS KO cells were maintained in Power CHO medium (Lonza, BELN12‐771Q) supplemented with 4 mm glutamine. The cells were maintained in 125‐mL Erlenmeyer flasks (Corning, 431143) with culture medium(30 mL) in a humidified shaking incubator (Adolf Kuhner AG, ISF1‐X) at 37 °C, 110 rpm, and 5% CO_2_.

### Plasmid Construction and Gene Sourcing

All clonings were performed using In‐Fusion Snap Assembly (Takara, 638948) and KLD enzyme mix (NEB, M0554S). Split inteins (gp41‐1, SspGyrB, and MjaKlbA) were synthesized (Integrated DNA Technologies) and cloned into expression plasmids. A comprehensive list of plasmids used in this study is provided in Table S1 (Supporting Information). The BsAb‐encoding genes and their modifications were obtained from previous studies.^[^ ^17^, ^18^ ^]^ Additionally, PiggyBac (PB) transposase was synthesized (Integrated DNA Technologies) and cloned into the pcDNA3.1/Hygro(+) plasmid. A transposon, including a 5′ ITR sequence (5′‐TTAACCCTAGAAAGATAGTCTGCGTAAAATTGACGCATG‐3′) and a 3′ ITR sequence (5′‐CATGCGTCAATTTTACGCATGATTATCTTTAACGTACGTCACAATATGATTATCTTTCTAGGGTTAA‐3′), was inserted at the respective ends of the expression cassette (Figure 5A and 6F).

### Transfection

One day prior to transfection, CHO‐GS KO cells were seeded at 1.0 × 10^5^ cells mL^−1^ in 125 mL Erlenmeyer flasks in SFM4Transfx‐293 medium (Hyclone, SH30860.02) supplemented with 4 mm glutamine. On the day of transfection, the cells were seeded at 1.0 × 10^6^ cells mL^−1^ in six‐well plates and transfected with the DNA–lipid complex formed by mixing 3 µg of plasmid and 6 µL of 293fectin (Thermo Fisher Scientific, 12347019) in 200 µL of Opti‐MEM (Thermo Fisher Scientific, 31985062).

To generate SiMPl‐GS‐based recombinant protein‐expressing pools, cells were transfected with N‐ and C‐terminal plasmids at a 1:1 ratio (w/w%). For transposon‐mediated gene transfer, cells were transfected with a transposon plasmid and transposase plasmid at a 3:1 ratio (w/w%).

### Cell Pool Generation

To generate cell pools expressing GOI, on the second‐day post‐transfection, transfected cells were transferred to 125‐mL Erlenmeyer flasks at 0.3 × 10^6^ cells mL^−1^ in selection medium (PowerCHO medium supplemented with GSEM; Sigma–Aldrich, G9785) 2 days after transfection and cultured until cell viability reached over 90%. Subsequently, they were subjected to further analysis.

### Flow Cytometry

mCherry and EGFP expression levels were measured in fluorescent protein‐expressing pools. For therapeutic protein‐producing cell pools, cell surface staining was performed as described previously.^[^ ^51^ ^]^ with a goat anti‐human IgG (gamma‐chain specific)‐R‐PE conjugate (Jackson ImmunoResearch, 109‐116‐098). Fluorescence intensity was measured using an LSR LSRFortessa (BD Biosciences) and analyzed using FlowJo software (BD Biosciences).

### Western Blot Analysis

Cells were lysed with Pro‐prep (iNtRon, 17081) according to the manufacturer's instructions. Total protein concentration was measured using a BCA Protein Assay Kit (Thermo Fisher Scientific, 23225). Subsequently, 20 µg of the lysed proteins were mixed with LDS Sample Buffer (Thermo Fisher Scientific, NP0008). The samples were heated at 95 °C for 10 min, after which sodium dodecyl‐sulfate polyacrylamide gel electrophoresis separation was conducted. Proteins were then transferred to polyvinylidene fluoride membranes using iBlot 2 (Thermo Fisher Scientific). Membranes were blocked with 5% skim milk dissolved in TBST for 1 h at room temperature. The membranes were then rinsed with TBST and incubated with the antibodies listed in Table S2 (Supporting Information). Target proteins were detected using the ChemiDoc software (Bio‐Rad).

### Measurement of Glutamine Concentration

The concentration of glutamine in the cell culture supernatant was determined using CedexBio (Roche) after harvesting the supernatant via centrifugation.

### Batch and Fed‐Batch Cultures for Recombinant Protein Production

For batch culture, cells were seeded at a concentration of 3 × 10^5^ cells mL^−1^ in 125‐mL Erlenmeyer flasks containing 30 mL of selection medium, either with or without MSX. The viable cell concentration and viability were measured using a Countess II FL automated cell counter (Thermo Fisher Scientific). Cell pellets and culture supernatants were stored at −70 °C for further analysis.

For fed‐batch culture, the cells were fed with BalanCD CHO Feed 3 (Irvine Scientific) at 5% working volume every other day from day 3, and the culture temperature was shifted to 33 °C on day 4.

The therapeutic protein concentrations in the culture supernatant were determined using enzyme‐linked immunosorbent assay (ELISA) and compensated by CedexBio. For ELISA, anti‐human IgG (Sigma–Aldrich, I1886) and anti‐human IgG‐peroxidase (Sigma–Aldrich, A0170) antibodies were used for coating and detection, respectively. Specific productivities, *q*_ETN_, *q*_mAb_, and *q*_BsAb_, were calculated from a plot of recombinant protein concentrations against the time‐integral values of the viable cell concentration.

### Quantitative Reverse Transcription‐Polymerase Chain Reaction (qRT‐PCR)

For mRNA expression analysis, total RNA was isolated from the cells using RiboEX and Hybrid‐R RNA isolation kits (GeneAll, 305‐101) according to the manufacturer's instructions. The concentration of the isolated RNA was quantified using a NanoDrop 2000 spectrophotometer (Thermo Fisher Scientific). Subsequently, cDNA was synthesized from 1 µg of RNA using a high‐capacity cDNA Reverse Transcription Kit (Thermo Fisher Scientific, 4368814). The synthesized cDNA was mixed with SYBR Green master mix (Bio‐Rad). The primers used are listed in Table S3 (Supporting Information).

For gene copy number analysis, gDNA was isolated from the cells using Exgene Blood SV (GeneAll, 305‐101) according to the manufacturer's instructions. The concentration of the isolated gDNA was determined using a NanoDrop 2000 spectrophotometer. 100 µg of gDNA were mixed with TaqMan Gene Expression Master Mix (Thermo Fisher Scientific, 4369016), along with the primers and probes listed in Table S4 (Supporting Information).

qRT‐PCR was performed using a CFX96 real‐time PCR detection system (Bio‐Rad), and the data were analyzed using the CFX Manager software (Bio‐Rad).

### Single‐Cell Cloning Using Beacon Instrument

Stable pools were loaded as single cells onto OptoSelect chips (Berkeley Lights, 750‐00018) using a Beacon optofluidic system (Berkeley Lights). Cells were cultured on the chips for 4 days, and the secretion assay was performed on day 4 using the Spotlight Human Fc assay reagent (Berkeley Lights, 520‐00024). The AU scores, which represent the secretion levels, were recorded for each pen and subsequently normalized using the AU score from an empty pen as a reference. Scores were magnified by a factor of 1000× for each pen and then filtered to include only data from pens with five or more cells. Subsequently, clonal productivity analysis was conducted on data from 280 randomly selected clones from each sample.

### Statistical Analyses

Data are presented as the mean ± standard deviation (*n* = 3 unless otherwise noted). The coefficient of determination (R^2^) was calculated using linear regression. Data were analyzed using a paired two‐tailed Student's *t*‐test, and significance was set at p < 0.05. Significant differences are indicated by *p < 0.05, **p < 0.01, ***p < 0.001, and ****p < 0.0001. NS, not significant; ND, not detected.

## Conflict of Interest

CY and GML have filed a patent application for the technology discussed in this paper through KAIST. The other authors declare no competing interests.

## Author Contributions

C.Y. and G.M.L. conceptualized the study. C.Y., D.K., and E.L. developed the methodology. C.Y., D.K., E.L., S.Y., Y.K., and H.J., performed the investigation. C.Y. visualized the study., Y.‐G.K., and G.M.L., supervised the study. C.Y. and G.M.L. wrote the original draft. C.Y., D.K., E.L., Y.‐G.K., and G.M.L. wrote, reviewed, and edited the final draft.

## Supporting information

Supporting Information

## Data Availability

The data that support the findings of this study are available from the corresponding author upon reasonable request.
