# Supplementary material for: SiMPl‐GS: Advancing Cell Line Development via Synthetic Selection Marker for Next‐Generation Biopharmaceutical Production
Source: Adv Sci (Weinh). 2024 Aug 6;11(38):2405593. doi: 10.1002/advs.202405593 (PMC11481413; doi:10.1002/advs.202405593)
Supplement: Supplementary file 1 — Supporting Information [file ADVS-11-2405593-s001.docx]

Supporting Information

______________________________________________________________________

**SiMPL-GS: Advancing cell line development via synthetic selection marker for next-generation biopharmaceutical production**

*Chansik Yoon, Eun-ji Lee, Dongil Kim, Siyun Joung, Yujin Kim, Heungchae Jung, Yeon-Gu Kim¸Gyun Min Lee**

C Yoon, D Kim, S Joung, Y Kim, G Lee

Department of Biological Sciences, KAIST, Daejeon 34141, Republic of Korea

Email: gyunminlee@kaist.ac.kr

E Lee, Y Kim,

Biotherapeutics Translational Research Center, KRIBB, Daejeon 34113, Republic of Korea

Department of Bioprocess Engineering, KRIBB School of Biotechnology, UST, Daejeon 34141, Republic of Korea

H Jung

BIO Center, Daejeon Technopark, Daejeon 34054, Republic of Korea

**This PDF file includes:**

Figures S1 to S9

Tables S1 to S4


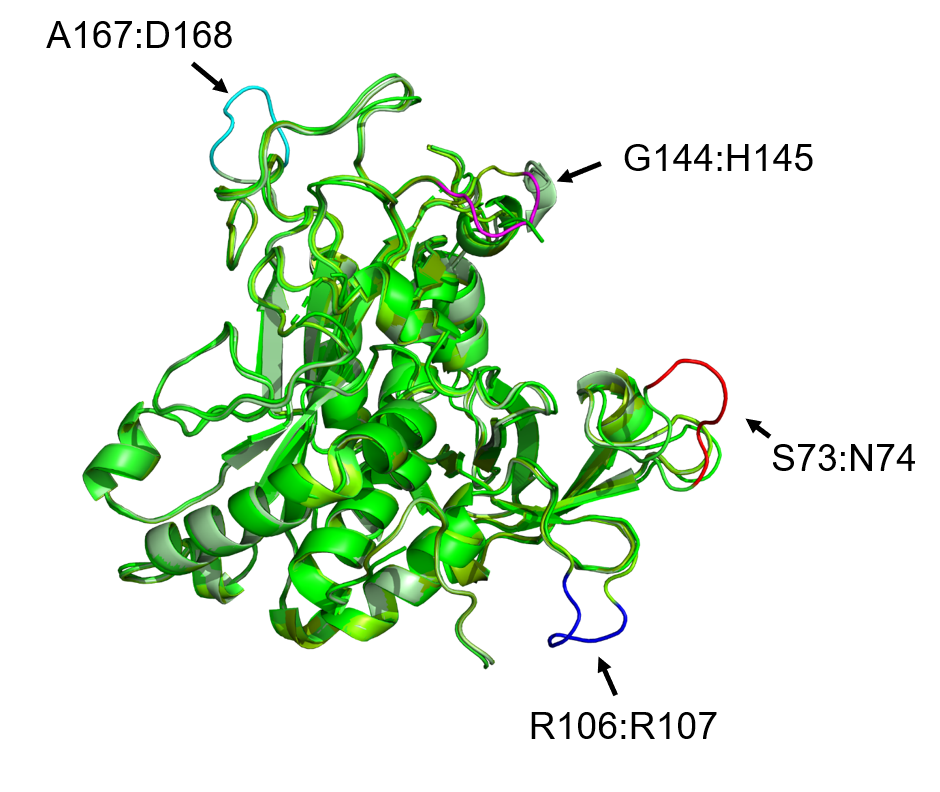


Figure S1. Predicted structure of trans-Spliced GS (PDB: 2OJW) for NC1, NC2, NC3, and NC4. The structure of trans-spliced GS was predicted using AlphaFold2 Colab. The alignment of proteins was accomplished with RaptorX. Distinct colorations represent the trans-spliced regions: red for NC1, blue for NC2, purple for NC3, and sky blue for NC4. Potential split sites were highlighted by black arrows.


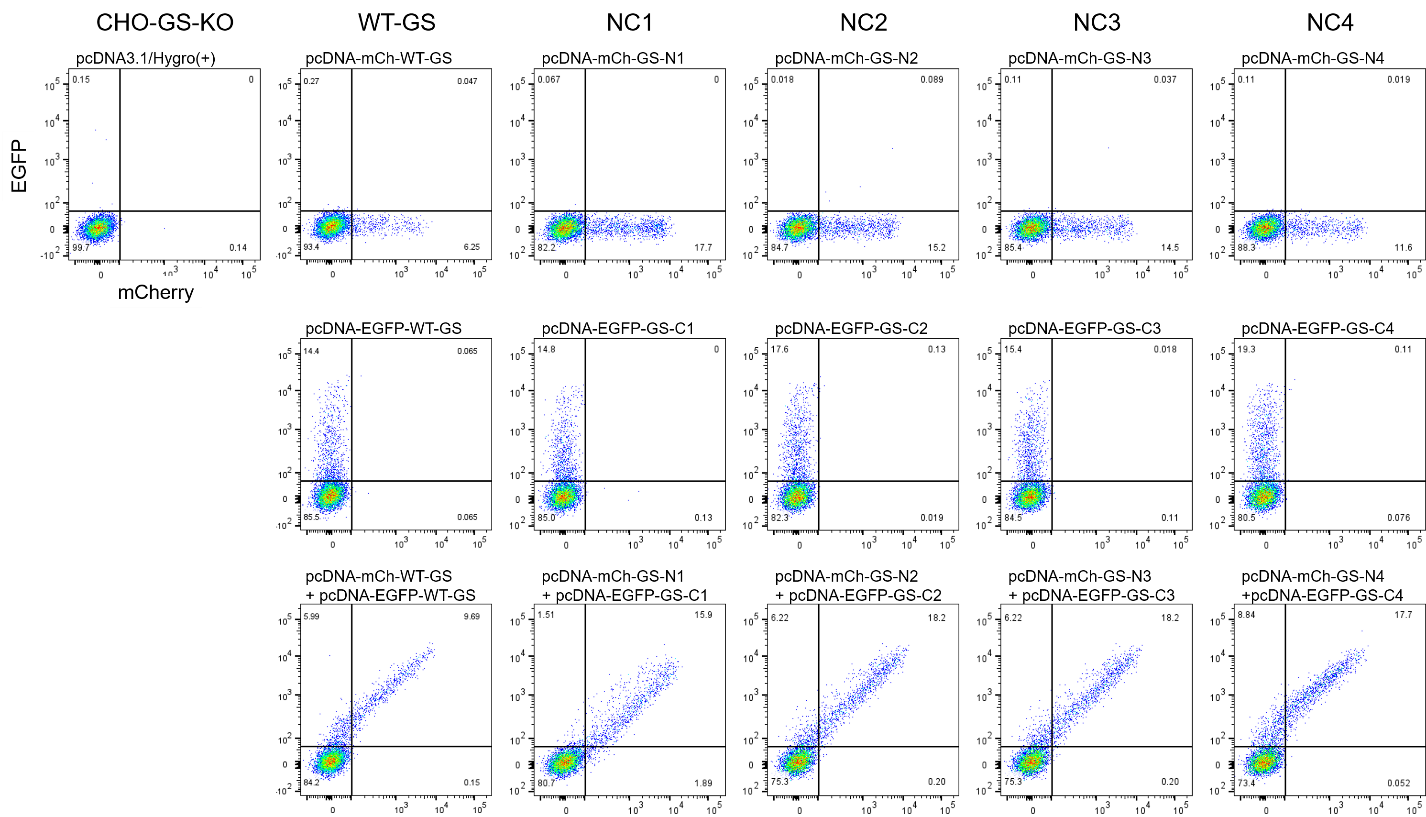


Figure S2. Distribution of fluorescence protein-expressing cells measured using flow cytometry. Cells were transfected with the indicated plasmids (see Supplementary Table 1), incubated for two days, and then analyzed using flow cytometry.


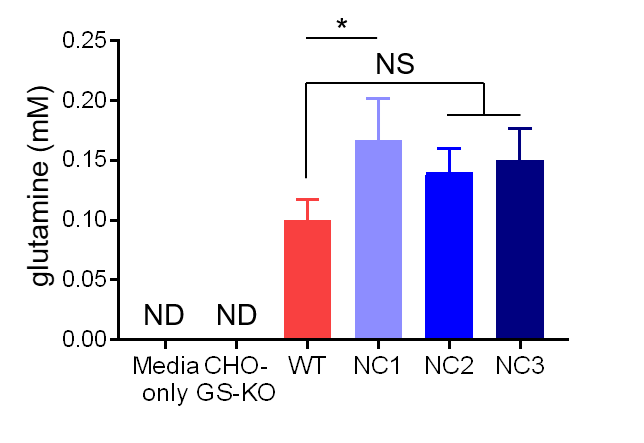


Figure S3. Glutamine concentrations in glutamine-free medium on day 3 (mean ± SD, n = 3). *p*-values were calculated by paired two-tailed Student’s *t*-test, NS, not significant; ND, not detected; **p* < 0.05.

**
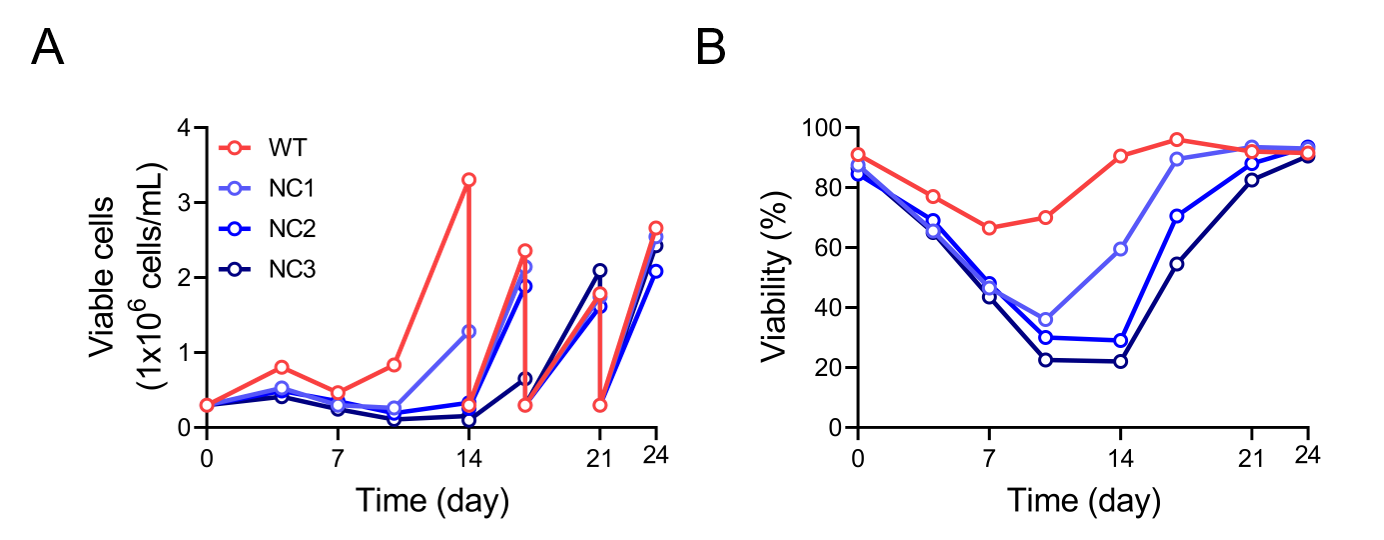
**

Figure S4. Selection and recovery profile of CHO-GS KO cells transfected with etanercept (ETN)-expressing plasmids (Supplementary Table 1). Transfected cells were cultured in glutamine-free medium in 125-mL Erlenmeyer flasks, and viable cells and viability were evaluated every three or four days. When viable cells reached over 0.1 x 10^6^ cells/mL, cells were passaged into new flasks until the viability reached over 90%.


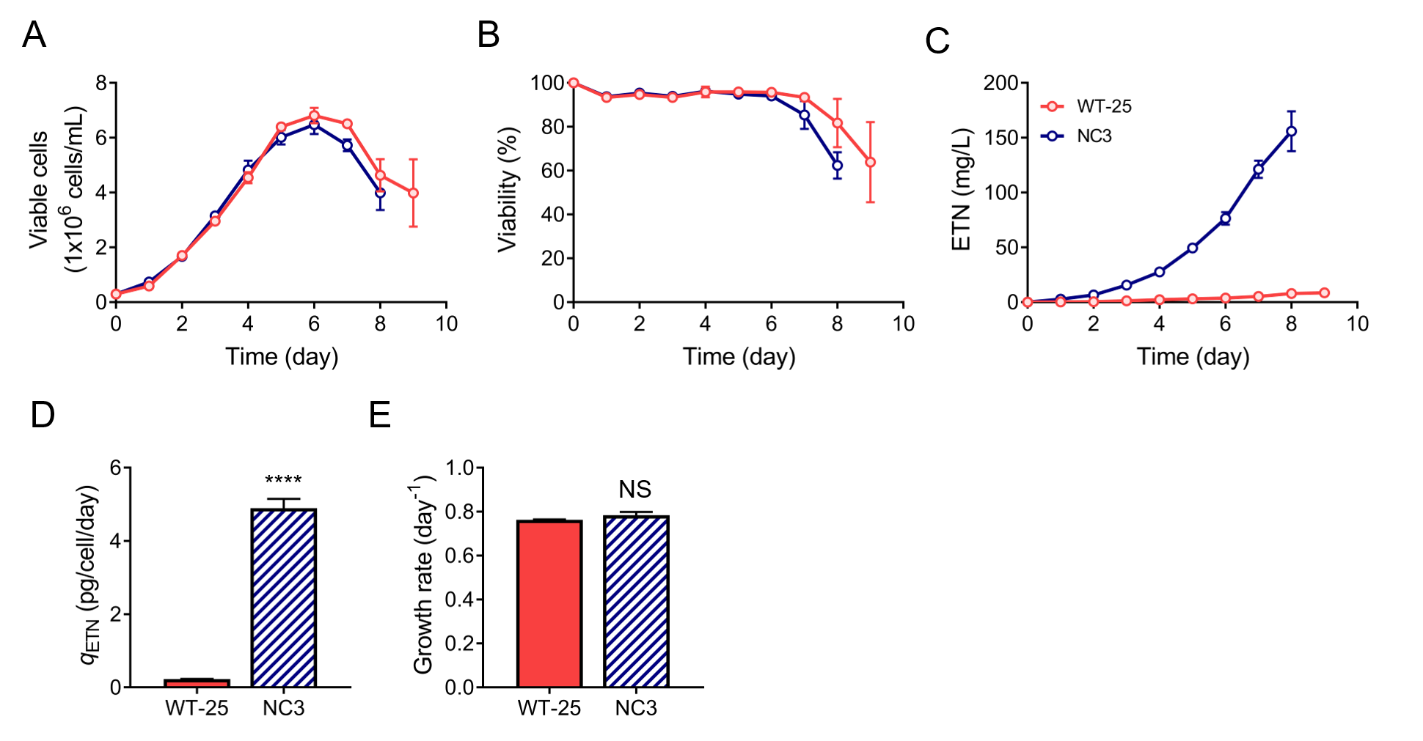


Figure S5. Culture profile of ETN-producing NC3 variant without MSX. (A-C) Profiles of cell growth (A), cell viability (B), and ETN concentration (C) (mean ± SD, *n* = 3). ETN-producing WT-GS cells cultured with 25 μM of MSX (WT-25) were used as control. (D) Specific productivity (*q*_ETN_) derived from (A) and (C) (mean ± SD, *n* = 3). (E) Specific growth rate (μ) calculated based on the data collected from day 0 to day 3 (mean ± SD, *n* = 3). *p*-values were calculated by paired two-tailed Student’s *t*-test, NS, not significant; *****p* < 0.0001.


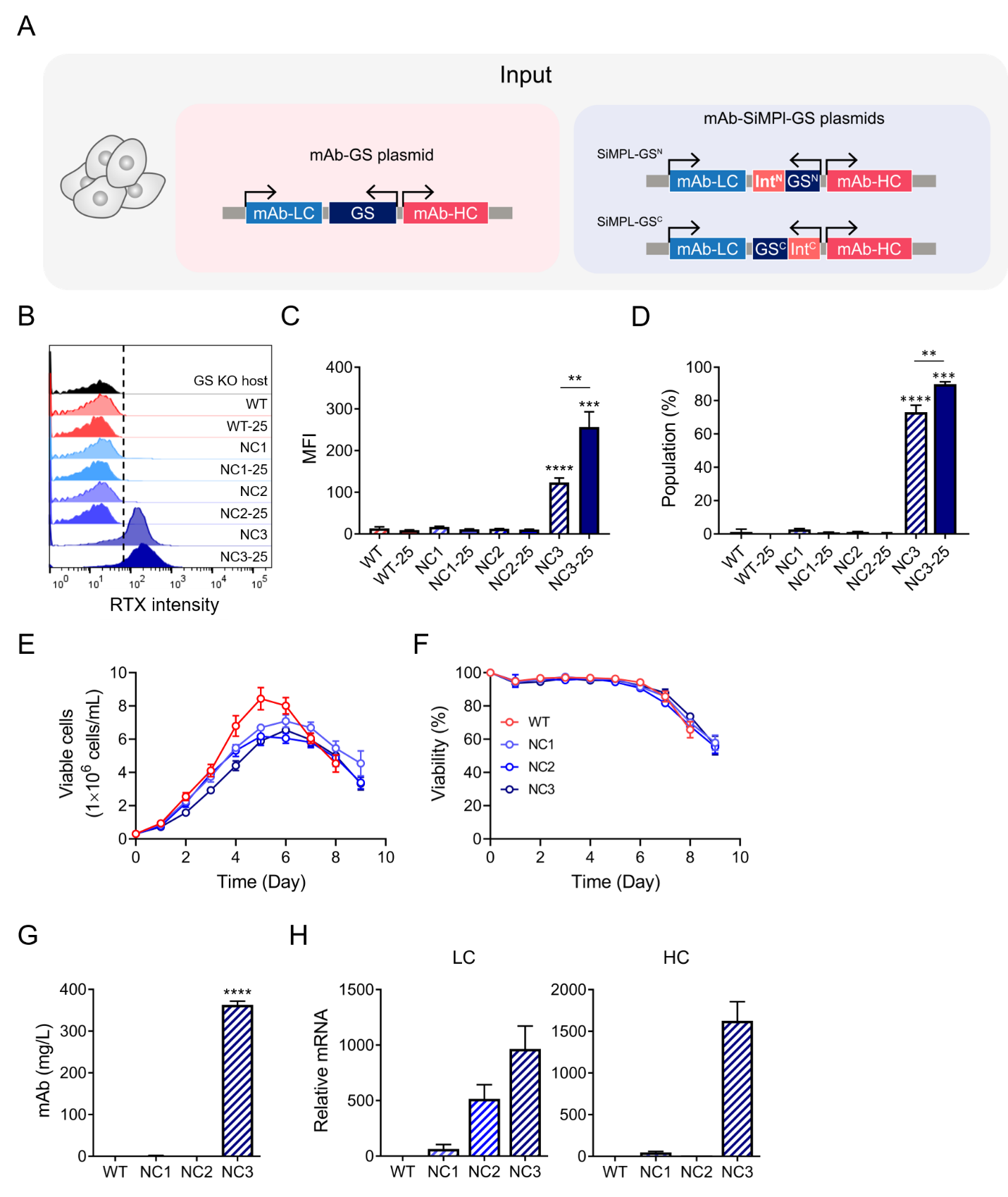


Figure S6. SiMPl-GS system for generating mAb-producing cells. (A) Schematic of mAb-producing WT-GS (left) and SiMPl-GS (right) plasmids. (B–D) Flow cytometric analysis of SiMPl-GS selection system for mAb-producing cell pools (mean ± SD, *n* = 3). Cells were further incubated with 25 μM MSX. Histogram (B), MFI, (C) and mAb-producing population (D) determined by staining mAb on the cell surface. (E and F) Profiles of cell growth (E) and viability (F) of mAb-producing cell pools (mean ± SD, *n* = 3). (G) mAb concentration of indicated cell pools measured on day 9 of culture (mean ± SD, *n* = 3). (H) Relative mRNA expression level of heavy chain (HC, left) and light chain (LC, right) (mean ± SD, *n* = 3). The mRNA level was normalized to *GAPDH*. *p*-values were calculated by paired two-tailed Student’s *t*-test, ***p* < 0.01; ****p* < 0.001; *****p* < 0.0001.


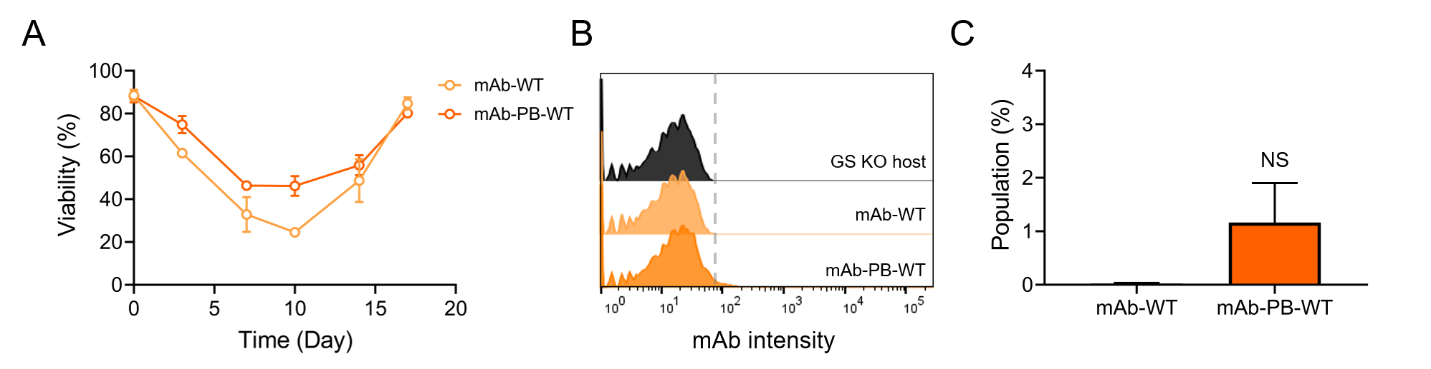


Figure S7. Transposon-based generation of cell pools. (A) Viability of mAb-producing cell pools (mean ± SD, *n* = 3). mAb-WT and mAb-PB-WT cell pools were generated by transfecting pcDNA-mAb-PB-WT-GS plasmid either with or without pcDNA-PB-transposase plasmid. Three independent cell pools were generated. (B and C) Flow cytometric analysis of transposon-based integration of indicated cell pools (mean ± SD, *n* = 3). Histogram (B) and mAb-producing population (C) were determined by staining mAb on the cell surface. *p*-values were calculated by paired two-tailed Student’s *t*-test, NS, not significant.

**
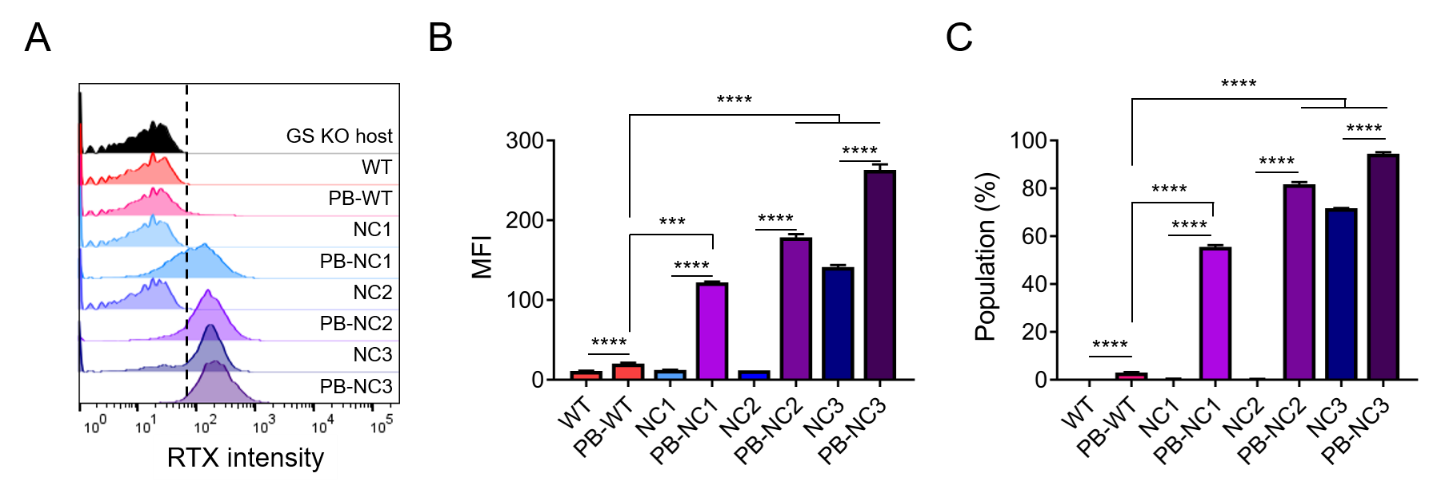
**

Figure S8. Flow cytometric analysis of the cooperative performance of transposon-based integration method and SiMPl-GS selection system for mAb-producing cell pools. Histogram (A), MFI (B) and mAb-producing population (C) determined by staining mAb on the cell surface (mean ± SD, *n* = 3). PB, piggybac transposon. *p*-values were calculated by paired two-tailed Student’s *t*-test, ****p* < 0.001; *****p* < 0.0001.

**
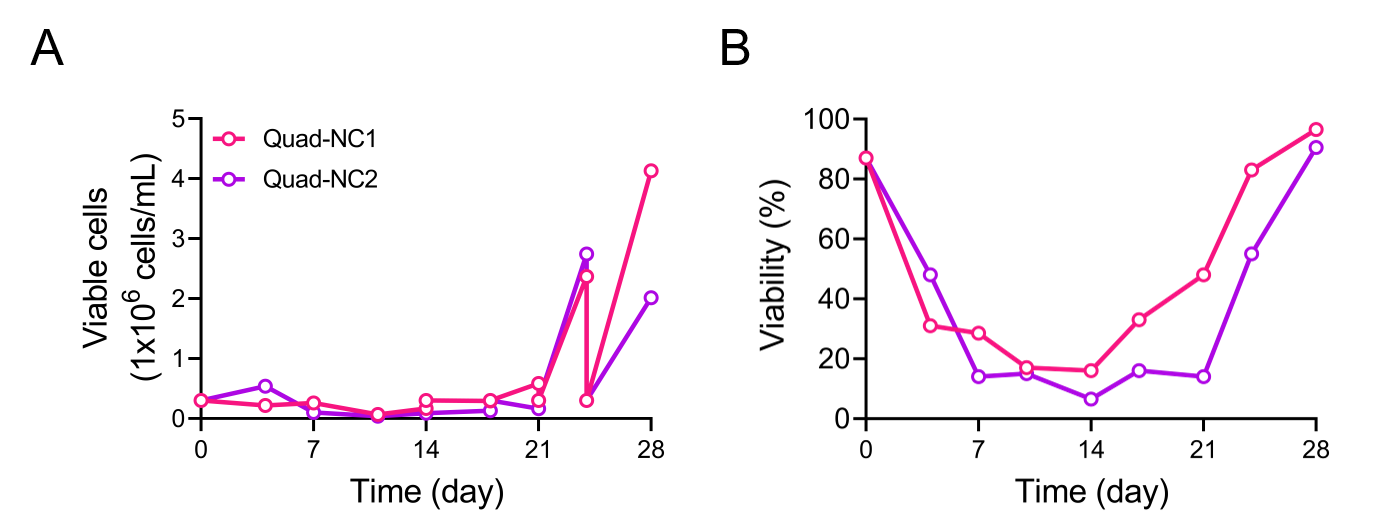
**

Figure S9. Selection and recovery profiles of CHO-GS KO cells transfected with BsAb-expressing quad plasmids (Supplementary Table. 1). Transfected cells were cultured in glutamine-free medium in 125 mL Erlenmeyer flasks, and viable cells and viability were estimated on every three or four days. When viable cell concentration reached over 0.1 x 10^6^ cells/mL, cells were passaged into new flasks. Cells were cultured until the viability reached over 90%.

Table S1. Plasmids used in the study.

| No. | Plasmid Name | Structure^a^ |
| --- | --- | --- |
| 1 | pcDNA3.1/Hygro(+) | Mammalian expression vector contains hygromycin |
| 2 | pcDNA-mCh-GS-WT | pCMV-mCherry-IRES-GS-WT |
| 3 | pcDNA-mCh-GS-N1 | pCMV-mCherry-IRES-GS^N1^-gp41-1^N^ |
| 4 | pcDNA-mCh-GS-N2 | pCMV-mCherry-IRES-GS^N2^-gp41-1^N^ |
| 5 | pcDNA-mCh-GS-N3 | pCMV-mCherry-IRES-GS^N3^-gp41-1^N^ |
| 6 | pcDNA-mCh-GS-N4 | pCMV-mCherry-IRES-GS^N4^-gp41-1^N^ |
| 7 | pcDNA-EGFP-GS-WT | pCMV-EGFP-IRES-GS-WT |
| 8 | pcDNA-EGFP-GS-C1 | pCMV-EGFP-IRES-gp41-1^C^-GS^C1^ |
| 9 | pcDNA-EGFP-GS-C2 | pCMV-EGFP-IRES-gp41-1^C^-GS^C2^ |
| 10 | pcDNA-EGFP-GS-C3 | pCMV-EGFP-IRES-gp41-1^C^-GS^C3^ |
| 11 | pcDNA-EGFP-GS-C4 | pCMV-EGFP-IRES-gp41-1^C^-GS^C4^ |
| 12 | pcDNA-mCh-GS-FLAG-N1 | pCMV-mCherry-IRES-FLAG-GS^N1^-gp41-1^N^ |
| 13 | pcDNA-mCh-GS-FLAG-N2 | pCMV-mCherry-IRES-FLAG-GS^N2^-gp41-1^N^ |
| 14 | pcDNA-mCh-GS-FLAG-N3 | pCMV-mCherry-IRES-FLAG-GS^N3^-gp41-1^N^ |
| 15 | pcDNA-mCh-GS-FLAG-N4 | pCMV-mCherry-IRES-FLAG-GS^N4^-gp41-1^N^ |
| 16 | pcDNA-EGFP-IRES-GS-HA-C1 | pCMV-EGFP-IRES-gp41-1^C^-GS^C1^-HA |
| 17 | pcDNA-EGFP-IRES-GS-HA-C2 | pCMV-EGFP-IRES-gp41-1^C^-GS^C2^-HA |
| 18 | pcDNA-EGFP-IRES-GS-HA-C3 | pCMV-EGFP-IRES-gp41-1^C^-GS^C3^-HA |
| 19 | pcDNA-EGFP-IRES-GS-HA-C4 | pCMV-EGFP-IRES-gp41-1^C^-GS^C4^-HA |
| 20 | pcDNA-ETN-GS-WT | pCMV-SVI-etanercept-GS-WT |
| 21 | pcDNA-ETN-GS-N1 | pCMV-SVI-etanercept-IRES-GS^N1^-gp41-1^N^ |
| 22 | pcDNA-ETN-GS-N2 | pCMV-SVI-etanercept-IRES-GS^N2^-gp41-1^N^ |
| 23 | pcDNA-ETN-GS-N3 | pCMV-SVI-etanercept-IRES-GS^N3^-gp41-1^N^ |
| 24 | pcDNA-ETN-GS-C1 | pCMV-SVI-etanercept-IRES-gp41-1^C^-GS^C1^ |
| 25 | pcDNA-ETN-GS-C2 | pCMV-SVI-etanercept-IRES-gp41-1^C^-GS^C2^ |
| 26 | pcDNA-ETN-GS-C3 | pCMV-SVI-etanercept-IRES-gp41-1^C^-GS^C3^ |
| 27 | pcDNA-singlemAb-HC-GS-N1 | pCMV-SVI-rituximab-HC-IRES-GS^N1^-gp41-1^N^ |
| 28 | pcDNA-singlemAb-LC-GS-C1 | pCMV-SVI-rituximab-LC-IRES-gp41-1^C^-GS^C1^ |
| 29 | pcDNA-singlemAb-HC-GS-C1 | pCMV-SVI-rituximab-HC-IRES-gp41-1^C^-GS^C1^ |
| 30 | pcDNA-singlemAb-LC-GS-N1 | pCMV-SVI-rituximab-LC-IRES-GS^N1^-gp41-1^N^ |
| 31 | pcDNA-singlemAb-HC-GS-N2 | pCMV-SVI-rituximab-HC-IRES-GS^N2^-gp41-1^N^ |
| 32 | pcDNA-singlemAb-LC-GS-C2 | pCMV-SVI-rituximab-LC-IRES-gp41-1^C^-GS^C2^ |
| 33 | pcDNA-singlemAb-HC-GS-C2 | pCMV-SVI-rituximab-HC-IRES-gp41-1^C^-GS^C2^ |
| 34 | pcDNA-singlemAb-LC-GS-N2 | pCMV-SVI-rituximab-LC-IRES-GS^N2^-gp41-1^N^ |
| 35 | pcDNA-singlemAb-HC-GS-N3 | pCMV-SVI-rituximab-HC-IRES-GS^N3^-gp41-1^N^ |
| 36 | pcDNA-singlemAb-LC-GS-C3 | pCMV-SVI-rituximab-LC-IRES-gp41-1^C^-GS^C3^ |
| 37 | pcDNA-singlemAb-HC-GS-C3 | pCMV-SVI-rituximab-HC-IRES-gp41-1^C^-GS^C3^ |
| 38 | pcDNA-singlemAb-LC-GS-N3 | pCMV-SVI-rituximab-LC-IRES-GS^N3^-gp41-1^N^ |
| 39 | pcDNA-mAb-GS-WT | pCMV-rituximab-LC-pSV40-GS-WT-pCMV-rituximab-HC |
| 40 | pcDNA-mAb-GS-N1 | pCMV-rituximab-LC-pSV40-GS^N1^-gp41-1^N^-pCMV-rituximab-HC |
| 41 | pcDNA-mAb-GS-C1 | pCMV-rituximab-LC-pSV40-GS^N2^-gp41-1^N^-pCMV-rituximab-HC |
| 42 | pcDNA-mAb-GS-N2 | pCMV-rituximab-LC-pSV40-GS^N3^-gp41-1^N^-pCMV-rituximab-HC |
| 43 | pcDNA-mAb-GS-C2 | pCMV-rituximab-LC-pSV40-gp41-1^C^-GS^C1^-pCMV-rituximab-HC |
| 44 | pcDNA-mAb-GS-N3 | pCMV-rituximab-LC-pSV40-gp41-1^C^-GS^C2^-pCMV-rituximab-HC |
| 45 | pcDNA-mAb-GS-C3 | pCMV-rituximab-LC-pSV40-gp41-1^C^-GS^C3^-pCMV-rituximab-HC |
| 46 | pcDNA-PB-transposase | pcDNA-piggybac transposase |
| 47 | pcDNA-mAb-PB-GS-WT | 5'ITR-pCMV-rituximab-LC-pSV40-GS-WT-pCMV-rituximab-HC-3'ITR |
| 48 | pcDNA-mAb-PB-GS-N1 | 5'ITR-pCMV-rituximab-LC-pSV40-GS^N1^-gp41-1^N^-pCMV-rituximab-HC-3'ITR |
| 49 | pcDNA-mAb-PB-GS-N2 | 5'ITR-pCMV-rituximab-LC-pSV40-GS^N2^-gp41-1^N^-pCMV-rituximab-HC-3'ITR |
| 50 | pcDNA-mAb-PB-GS-N3 | 5'ITR-pCMV-rituximab-LC-pSV40-GS^N3^-gp41-1^N^-pCMV-rituximab-HC-3'ITR |
| 51 | pcDNA-mAb-PB-GS-C1 | 5'ITR-pCMV-rituximab-LC-pSV40-gp41-1^C^-GS^C1^-pCMV-rituximab-HC-3'ITR |
| 52 | pcDNA-mAb-PB-GS-C2 | 5'ITR-pCMV-rituximab-LC-pSV40-gp41-1^C^-GS^C2^-pCMV-rituximab-HC-3'ITR |
| 53 | pcDNA-mAb-PB-GS-C3 | 5'ITR-pCMV-rituximab-LC-pSV40-gp41-1^C^-GS^C3^-pCMV-rituximab-HC-3'ITR |
| 54 | pcDNA-ETN-Npu-GS-N1 | pCMV-SVI-etanercept-IRES-GS^N1N^puSsp^N^ |
| 55 | pcDNA-ETN-Npu-GS-N2 | pCMV-SVI-etanercept-IRES-GS^N2N^puSsp^N^ |
| 56 | pcDNA-ETN-Npu-GS-C1 | pCMV-SVI-etanercept-IRES^N^puSsp^C^-GS^C1^ |
| 57 | pcDNA-ETN-Npu-GS-C2 | pCMV-SVI-etanercept-IRES^N^puSsp^C^-GS^C2^ |
| 58 | pcDNA-ETN-Cth-GS-N1 | pCMV-SVI-etanercept-IRES-GS^N1C^th-Ter^N^ |
| 59 | pcDNA-ETN-Cth-GS-N2 | pCMV-SVI-etanercept-IRES-GS^N2C^th-Ter^N^ |
| 60 | pcDNA-ETN-Cth-GS-C1 | pCMV-SVI-etanercept-IRES^C^th-Ter^C^-GS^C1^ |
| 61 | pcDNA-ETN-Cth-GS-C2 | pCMV-SVI-etanercept-IRES^C^th-Ter^C^-GS^C2^ |
| 62 | pcDNA-ETN-SspGyrB-GS-N1 | pCMV-SVI-etanercept-IRES-GS^N1^-SspGyrB^N^ |
| 63 | pcDNA-ETN-SspGyrB-GS-N2 | pCMV-SVI-etanercept-IRES-GS^N2^-SspGyrB^N^ |
| 64 | pcDNA-ETN-SspGyrB-GS-C1 | pCMV-SVI-etanercept-IRES-SspGyrB^C^-GS^C1^ |
| 65 | pcDNA-ETN-SspGyrB-GS-C2 | pCMV-SVI-etanercept-IRES-SspGyrB^C^-GS^C2^ |
| 66 | pcDNA-ETN-MjaKlbA-GS-N1 | pCMV-SVI-etanercept-IRES-GS^N1^-MjaKlbA^N^ |
| 67 | pcDNA-ETN-MjaKlbA-GS-N2 | pCMV-SVI-etanercept-IRES-GS^N2^-MjaKlbA^N^ |
| 68 | pcDNA-ETN-MjaKlbA-GS-C1 | pCMV-SVI-etanercept-IRES-MjaKlbA^C^-GS^C1^ |
| 69 | pcDNA-ETN-MjaKlbA-GS-C2 | pCMV-SVI-etanercept-IRES-MjaKlbA^C^-GS^C2^ |
| 70 | pcDNA-mCh-N-gp41-1-N | pCMV-SVI-mCherry^N^-gp41-1^N^ |
| 71 | pcDNA-mCh-N-SspGyrB-N | pCMV-SVI-mCherry^N^-SspGyrB^N^ |
| 72 | pcDNA-mCh-N-MjaKlbA-N | pCMV-SVI-mCherry^N^-MjaKlbA^N^ |
| 73 | pcDNA-gp41-1-C-mCh-C | pCMV-SVI-gp41-1^N^-mCherry^C^ |
| 74 | pcDNA-SspGyrB-C-mCh-C | pCMV-SVI-SspGyrB^N^-mCherry^C^ |
| 75 | pcDNA-MjaKlbA-C-mCh-C | pCMV-SVI-MjaKlbA^N^-mCherry^C^ |
| 76 | pcDNA-PB-quad1-N1 | 5'ITR-pCMV-SVI-pertuzumab-LC-IRES-GS^N1^-gp41-1^N^-3'ITR |
| 77 | pcDNA-PB-quad1-N2 | 5'ITR-pCMV-SVI-pertuzumab-LC-IRES-GS^N2^-gp41-1^N^-3'ITR |
| 78 | pcDNA-PB-quad2 | 5'ITR-pCMV-SVI-matuzumab-LC-IRES-gp41-1^C^-SspGyrB^N^-3'ITR |
| 79 | pcDNA-PB-quad3 | 5'ITR-pCMV-SVI-pertuzumab-HC-IRES-SspGyrB^C^-MjaKlbA^N^-3'ITR |
| 80 | pcDNA-PB-quad4-C1 | 5'ITR-pCMV-SVI-matuzumab-HC-IRES-MjaKlbA^N^-GS^C1^-3'ITR |
| 81 | pcDNA-PB-quad4-C2 | 5'ITR-pCMV-SVI-matuzumab-HC-IRES-MjaKlbA^N^-GS^C2^-3'ITR |

^a^ SVI, SV40 intron; HC, heavy chain; LC, light chain; ITR, inverted terminal repeat.

Table S2. Antibodies used for western blot analysis.

| Antibody | Host | Company | Catalog |
| --- | --- | --- | --- |
| Anti-FLAG | Mouse | Merck | #F1804 |
| Anti-HA | Mouse | Cell Signaling Technology | #3724 |
| Anti-GS | Mouse | Abcam | #ab135622 |
| Anti-EGFP | Rabbit | Abcam | #ab290 |
| Anti-mCherry | Rabbit | Abcam | #167453 |
| Anti-β-actin | Mouse | Cell Signaling Technology | #4967 |
| Anti-mouse IgG, HRP-linked antibody | Rabbit | Cell Signaling Technology | #7076 |
| Anti-rabbit IgG, HRP-linked antibody | Mouse | Cell Signaling Technology | #7074 |

Table S3. Sequences of the primers for mRNA expression analysis.

| Primer set name | Forward primer (5′→3′) | Reverse primer (5′→3′) |
| --- | --- | --- |
| *GAPDH* | GGACATCAAGAAGGTGGTGAA | GAGTGGGAGTCACTGTTGAAG |
| ETN | CCAGACCAGGAACTGAAACAT | GGATGAAGTCGTGTTGGAGAA |
| mAb-HC | CAGCCGGAGAACAACTACAA | CATCACGGAGCATGAGAAGA |
| mAb-LC | GTTGTGTGCCTGCTGAATAAC | TCCTGCTCTGTGACACTCT |

Table S4. Primers and probes used for gene copy number analysis.

| Primer set name | Forward primer (5′→3′) | Probe (5′→3′) | Reverse primer (5′→3′) |
| --- | --- | --- | --- |
| *C1GALT1C1* | ACCCGAACCAGGTAGTAGAA | AGTGACAGCCATATTGGAACAGCATCC | ACATGTCCAAAGGCCCTAAG |
| EGFP | GCACAAGCTGGAGTACAACTA | TGTTGTGGCGGATCTTGAA | TGTTGTGGCGGATCTTGAA |
| mCherry | GACTACTTGAAGCTGTCCTTCC | TTCAAGTGGGAGCGCGTGATGAA | CGCAGCTTCACCTTGTAGAT |
| ETN | CAGCCGGAGAACAACTACAA | TACAGCAAGCTCACCGTGGACAAG | CATCACGGAGCATGAGAAGA |
| mAb-HC | CAGCCGGAGAACAACTACAA | TACAGCAAGCTCACCGTGGACAAG | CATCACGGAGCATGAGAAGA |
| mAb-LC | CTGCACCATCTGTCTTCATCT | TCTGGAACTGCCTCTGTTGTGTGC | GCCTCTCTGGGATAGAAGTTATTC |
